# Microfilariae in a bone marrow aspirate

**DOI:** 10.1186/s13104-016-2051-1

**Published:** 2016-05-04

**Authors:** Santosh Tummidi, Manoj Kumar Patro, Atanu Kumar Bal, Anita Choudhury

**Affiliations:** Department of Pathology and Lab Medicine, AIIMS, Raipur, Chhattisgarh India; Department of Pathology, MKCG Medical College, Berhampur, Odisha India

**Keywords:** Microfilariae, Bone marrow, Filariasis, Metastatic deposit

## Abstract

**Background:**

Filariasis is a common cause of morbidity in certain parts of India, especially in the Coastal Districts. Repeated episodes of fever with chills and rigor, lymphadenopathy are the initial manifestations which gradually progress to elephantiasis. *Wuchereria bancrofti* is the most common parasite causing lymphatic filariasis in India. Detecting microfilaria in peripheral blood with or without *Diethylcarbamazine* citrate provocation is the common diagnostic modality in suspected cases. However microfilaria has been accidentally detected in fine needle aspirates, aspirated body fluids and even in bronchial washings.

**Case presentation:**

We report a case of 65-year old female presented with back ache. On investigation she had leuco-erythroblastic blood picture in the peripheral smear and metastatic deposits in the bone marrow aspirate. *W. bancrofti* microfilariae was an incidental finding in the bone marrow aspirate.

**Conclusion:**

Finding of microfilariae in the bone marrow aspirate in the absence of clinical features of lymphatic filariasis is extremely uncommon and mostly are incidental findings. The peripheral blood may or may not reveal the microfilariae and eosinophilia in the peripheral blood is absent in majority of the cases. All the bone marrow aspirates must be screened for microfilariae in the endemic areas to identify the asymptomatic carriers.

## Background

Filariasis has been a disabling parasitic disease world-wide particularly in tropical and subtropical countries of the world. The disease being fairly endemic all over India with causative agent being two closely related nematode worms, *Wuchereria bancrofti* and *Brugia malayi* and are transmitted by the female *Culex* mosquito [1]. These nematodes belong to the order Spirurida and superfamily Filarioidia. Microfilariae have been accidentally detected in the fine needle aspirate smears (FNAC) from thyroid, breast, subcutaneous nodules, cervical scraps, bronchial washings including body fluids [2]. The presence of microfilariae in bone marrow aspirate is an unusual finding. Association of filarial parasite with malignancy has been described but its role in tumorigenesis is not so far explained. We report a rare case of metastatic breast cancer to bone marrow with incidental finding of microfilaria.

## Case presentation

A 65 year-old-female, resident of Ganjam district was admitted to MKCG Medical College and Hospital with complains of back pain and weakness since 1 year. She was of average socio-economic status. Significant past history includes infiltrative duct carcinoma of left breast not otherwise specified category, operated 3½ years back. The immunohistochemical profile of the tumor was positive for both ER and PR, membrane negativity for Her-2/neu.

On examination, she had tenderness at 1st and 2nd lumbar vertebral region. Left mastectomy scar is healthy. No right breast mass lesion and no palpable axillary lymphadenopathy detected. Other systemic examinations revealed no abnormality. Routine hematological and biochemical investigations were normal except moderate anemia (7.4 gm/dL), Erythrocyte sedimentation rate (ESR) 30 mm in 1st hour. Peripheral smear showed leuco-erythroblastic blood picture. No hemoparasite was noted in the smear. Other significant investigation findings include multiple vertebral osteolytic lesions in the chest x-ray and mild hepatomegaly with multiple hypo-echoic lesions in ultrasonography (USG) of liver. Clinical impression was of metastatic lesion/multiple myeloma.

**Fig. 1:**
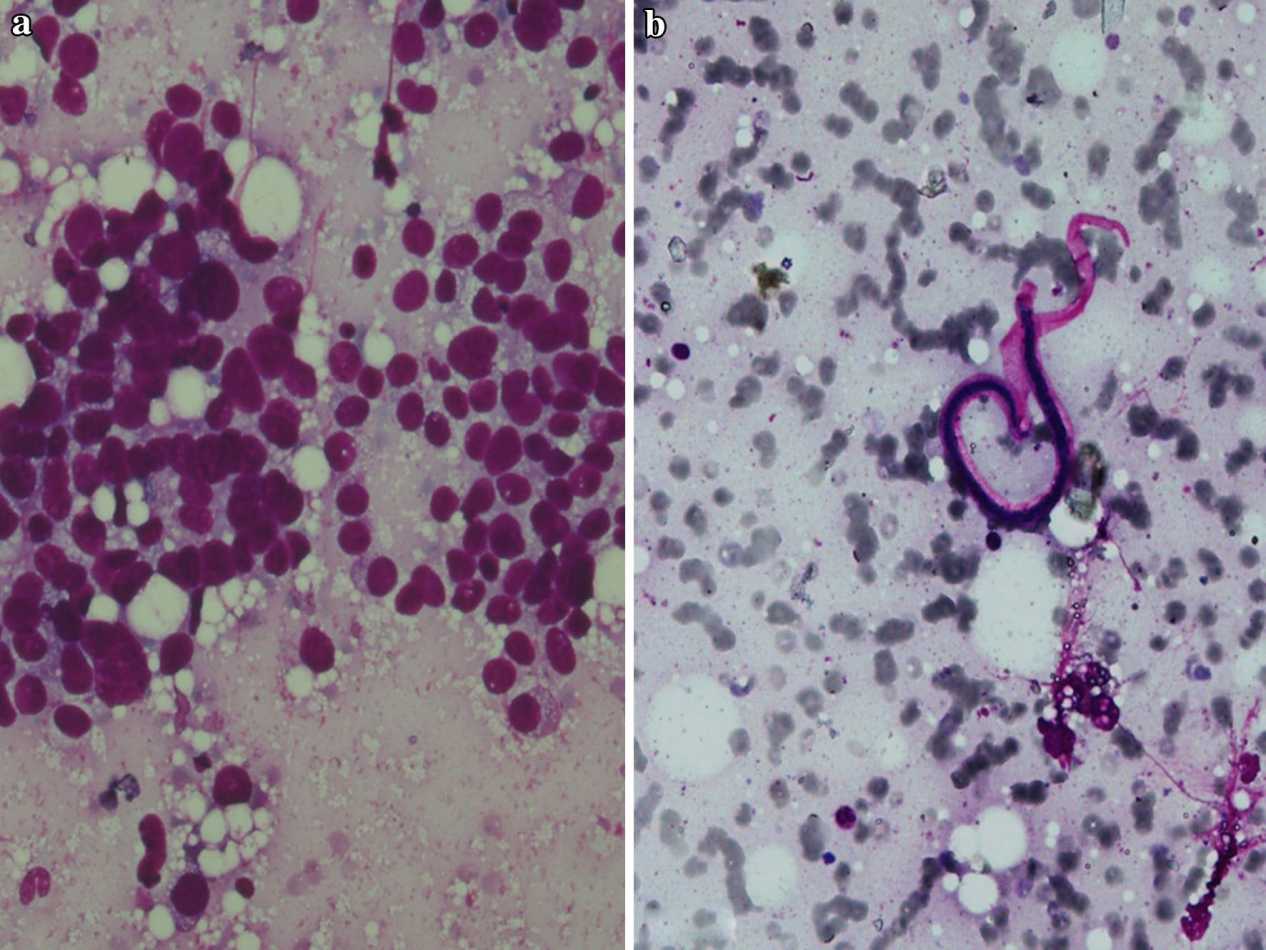
**a** Bone marrow aspirate (BMA) smear showing groups of metastatic tumor cells, looking round to oval with hyperchromatic nuclei and moderate amount of cytoplasm with cytoplasmic vacoulations. (Leishman, ×400); **b** BMA smear showing Microfilariae of *Wuchereria bancrofti* (Leishman, ×400)

**Fig. 2:**
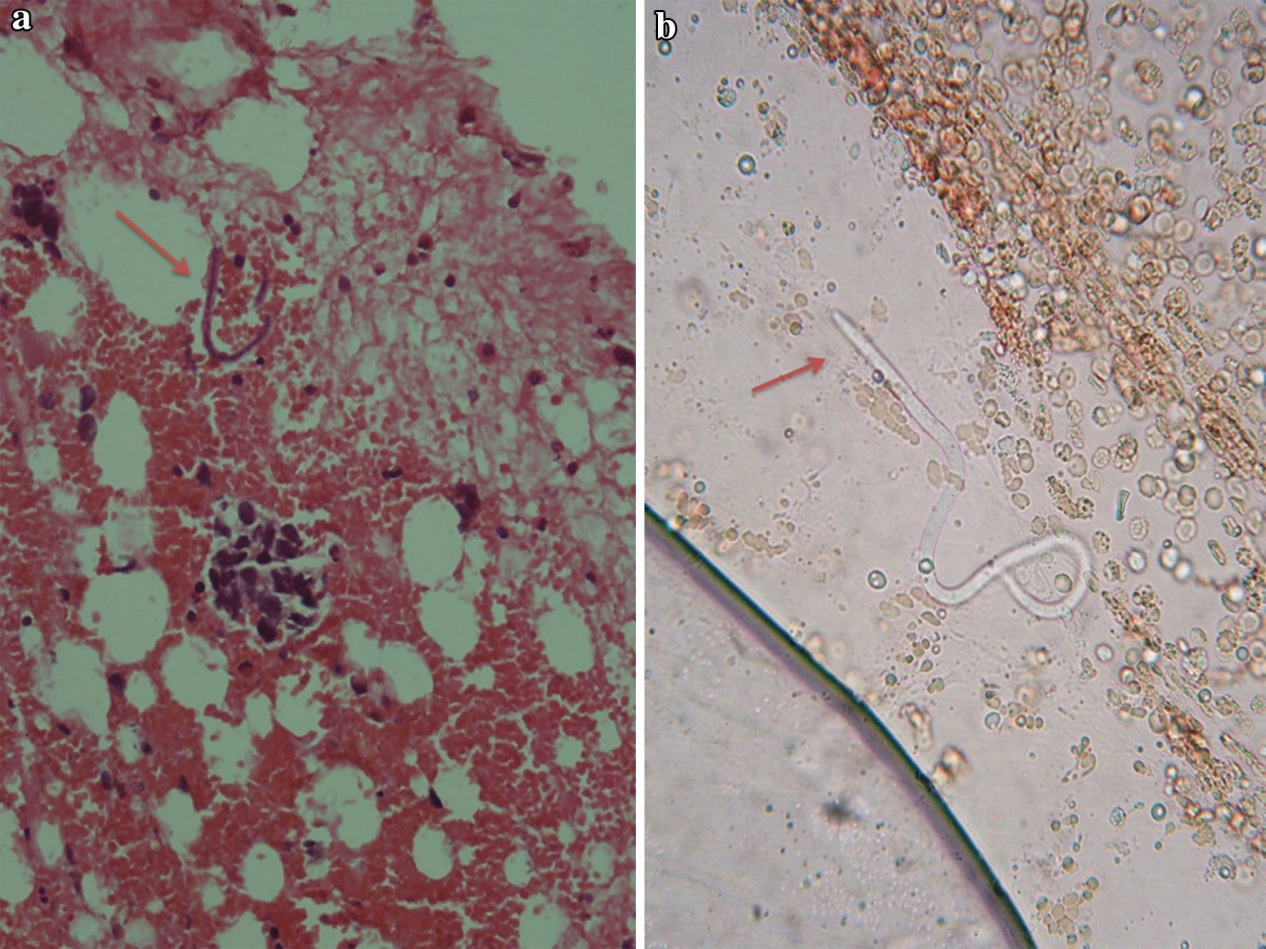
**a** Cell block of BMA showing Microfilariae along with metastatic tumor cell (HandE, ×400); **b** Wet mount of BMA showing Microfilariae. (×400)

Bone marrow aspirate and cell block sections showed suppression of the three cell lineage (i.e. erythroid, myeloid, megakaryocyte) with presence of pleomorphic tumor cells in acinar pattern, sheets and three dimensional clusters suggestive of adenocarcinomatous deposits. Tumor cells were round to oval with hyperchromatic nuclei and moderate amount of cytoplasm with cytoplasmic vacoulations (Fig. 1). A diagnosis of metastatic deposit in the bone marrow was made. Microfilariae of *Wuchereria bancrofti* were incidentally detected in aspirate smears, cell block and even in the wet mount of aspirate (Figs. 1a, 2a, b). Trephine biopsy impression was concordant with aspiration diagnosis. Immunohistochemical staining was done on trephine biopsy tissue for breast markers which showed that the tumor cells are negative for estrogen receptor (ER) and progesterone receptor (PR), but showed strong membrane positivity for Her-2/neu. Based on these findings the diagnosis of Metastatic breast carcinoma to bone with an incidental finding of *Wuchereria bancrofti* microfilariae was given.

## Discussion

In India filarial infestation is commonly caused by *W. bancrofti* and *Brugia malayi*, the former responsible for almost 98 % of all cases. Human beings serve as the definitive host for the parasite and mosquitoes serve as the intermediate host. In the definitive host i.e. in humans, the adult worm lodges in the lymphatics. The adult female parasite is ovoviviparous and gives birth to ova containing microfilariae that circulate in the blood stream [3]. The clinical spectrum of lymphatic filariasis ranges from only peripheral blood eosinophilia to lymphangitis finally terminating in elephantiasis. The exact mechanism regarding how these microfilariae come to extravascular tissue spaces is not known. The probable explanation being microfilariae in microcirculation cross the vessel wall by their boring ability and reach the tissue spaces. Hence they are picked up accidentally during aspiration from various sites [4]. They are found in the FNAC smears from the thyroid, breast, lymph nodes, sub-cutaneous nodules, in cervical scrape smears, in bronchial washings and in body fluids [2, 5].

The first documentation of microfilariae in bone marrow aspirate, available in English literature was by Pradhan et al. in 1976 [4]. Including the present case, none of the published cases where microfilariae was found in the bone marrow aspirates, had a classical clinical presentation of lymphatic filariasis [5–9]. A diagnosis of filariasis was made after demonstrating the microfilariae in the bone marrow aspirate or in the peripheral blood smears. In a few reported cases, microfilariae were present in the bone marrow aspirates but not in the peripheral blood [6, 9, 10]. The microfilariae of *W. bancrofti* is the most common type of parasite that is demonstrated in the bone marrow.

Peripheral blood eosinophilia is a common haematological finding in filariasis. But in a majority of the reported cases in which microfilariae were demonstrated in the bone marrow, eosinophilia was absent [4, 6, 7]. A similar finding of no blood eosinophilia was noted in our case. The bone marrow hemopoietic status in cases of microfilariae detected in marrow aspirates varied greatly from aplastic/hypoplastic marrow to hyperplastic marrow with a normoblastic or amegaloblastic maturation [4, 6, 7, 9]. The probable pathogenesis of marrow changes in these cases were attributed to other etiologic agents like drugs, viral infections, metastatic deposit or idiopathic and are unrelated to filarial infestation [6, 10, 11]. Filariasis can be cured by administration of Diethylcarbamazine citrate (DEC) [3].

## Conclusion

We consider that the presence of microfilariae in the bone marrow is an incidental finding and that filariasis has no role in the causation of metastatic deposit. The rarity of incidental detection of Microfilariae with a metastatic lesion in bone marrow has prompted us to report this case. In the endemic areas, all the bone marrow aspirates must be screened for microfilariae, to detect any asymptomatic carriers. Apart from DEC she needs the treatment for malignancy.

## Abbreviations

BMA: bone marrow aspirate
DEC: diethylcarbamazine citrate
ESR: erythrocyte sedimentation rate
FNAC: fine needle aspiration cytology
HandE: haematoxylin and eosin
USG: ultrasonography
